
JOURNAL INFORMATION
==============================
NLM Title Abbreviation: Tex Med J (Austin)
NLM Journal ID: 21130590R

Texas Medical Journal

ISSN: 0892-8495
Publisher: Biomedical Journal Digitization Project

ARTICLE INFORMATION
==============================
PMCID: PMC9590920
Article version: 1
Subjects: Books and Magazines

Urinary Analysis and Diagnosis, by Microscopical and Chemical Examinations

Print publication date: 1899 Jun
Volume: 14
Issue: 12
First page: 722
Last page: 722
License: This work is free of known copyright restrictions. For more information, please see PMC Back Issue Digitization.
License URL: https://creativecommons.org/publicdomain/mark/1.0/

==============================

---

Urinary Analysis and Diagnosis, by Microscopical and Chemical Examinations. By Louis Heitzmann, M. D., New York. One volume, 270 pp., 8vo. One hundred and eight original illustrations. Price, muslin, $2.00 net. William Wood & Co., Publishers, New York.

The first part of this work is devoted to chemical examination of the urine, the second to microscopical examination, and the third to microscopic diagnosis. The aim of the author has been to put forward a book for the busy practitioner, one free from impracticable tests and deductions. The subject is handled in a plain manner and the knowledge can be utilized by a physician of meagre attainments.

At this day the general practitioner must live more in the laboratory. He must do a great deal more himself, and it is possible, by the aid of such a volume as the one under consideration, for an average up to date doctor to avail himself of the advantage of the microscope and chemistry in his everyday work.

T. J. B.

---

### Publisher's Notes.

The Sterilization of Milk.—Shakespeare has wisely said "To gild refined gold, to paint the lily; to add fresh perfume to the violet is wasteful and ridiculous excess."

---

I have used Peacock's Chionia and find it very effective. I shall continue to prescribe it in my practice.

New York. A. P. Dalrymple, M. D.

---

"I have given your Neurosine (Dios) a thorough trial in a severe case of neurosis with tachycardia, and it has given entire satisfaction. In a case of epilepsy which I had in my outdoor clinic, the results, so far, are excellent.

C. H. Holzhausen, M. D.,
No. 352 West 21st Street, New York City.

---

Saunders' Medical Hand Atlases. The success of this series
